# Vitamin D status and prevalence of hypovitaminosis D in different genders throughout life stages: A Brazilian cross-sectional study

**DOI:** 10.6061/clinics/2021/e2571

**Published:** 2021-03-30

**Authors:** Lenora M. Camarate S.M. Leão, Bernardo Campos Rodrigues, Paulo Telles Pires Dias, Bárbara Gehrke, Thiago da Silva Pereira de Souza, Caio Kenji Hirose, Mônica Di Calafiori Freire

**Affiliations:** I Servico de Endocrinologia, Hospital Universitario Pedro Ernesto, Faculdade de Ciencias Medicas, Universidade do Estado do Rio de Janeiro, Rio de Janeiro, RJ, BR; II Faculdade de Ciencias Medicas, Universidade do Estado do Rio de Janeiro, Rio de Janeiro, RJ, BR; III Nucleo de Estudos e Pesquisas em Atencao ao Uso de Drogas, Universidade do Estado do Rio de Janeiro, Rio de Janeiro, RJ, BR; IV Programa de Pos-Graduacao em Fisiopatologia Clinica e Experimental (FISCLINEX), Faculdade de Ciencias Medicas, Universidade do Estado do Rio de Janeiro, Rio de Janeiro, RJ, BR; V Diagnosticos da America S/A, Sao Paulo, SP, BR; VI Sergio Franco Medicina Diagnostica, Rio de Janeiro, RJ, BR; VII Departamento de Epidemiologia e Bioestatistica (MEB-ISC), Universidade Federal Fluminense, Niteroi, RJ, BR

**Keywords:** 25-Hydroxivitamin D, Vitamin D Deficiency, Hypovitaminosis D Prevalence, Rio De Janeiro, Brazil

## Abstract

**OBJECTIVES:**

To evaluate the mean concentration of 25-hydroxivitamin D [25(OH) D] and prevalence of hypovitaminosis D in individuals residing in Rio de Janeiro, Brazil.

**METHODS:**

The data of 80,000 consecutive individuals who had 25(OH) D measurements performed by electrochemiluminescence between 1/2/2018 and 2/5/2018 were selected. Patients who reported the use of therapies/supplements were excluded. Levels of 25(OH) D ≥20 ng/mL (ages <60 years) and ≥30 ng/mL (ages ≥60 years) were considered adequate.

**RESULTS:**

We analyzed the data of 24,074 individuals (1-95 years old, 64.7% female). Descriptive curves showed that, in both sexes, the mean values of 25(OH) D decreased from the first years of life until adolescence, then slightly increased, and then tended to stabilize during adulthood. Levels of 25(OH) D <20 ng/mL were observed in 6% of girls *versus* 3.6% of boys and in 13.6% of adolescent girls *versus* 12.6% of adolescent boys and 11% of adults. The percentage of seniors with serum levels of 25(OH) D <20 ng/mL was 13.6% in women and 12.7% in men; 53.2% of women and 50.6% of men had levels <30 ng/mL.

**CONCLUSIONS:**

Mean 25(OH) D values were higher in children and lower in adolescents and women. Approximately 90% of non-seniors and presumably healthy residents of the urban metropolitan region of Rio de Janeiro presented satisfactory levels of 25(OH) D during the summer months; however, in over half of the elderly, the serum concentrations of 25(OH) D were inadequate. Therefore, strategies for the prevention of hypovitaminosis D should be considered in the senior population.

## INTRODUCTION

Vitamin D is a fat-soluble hormone that is present in two main forms (ergocalciferol and cholecalciferol); while it may be obtained through diet, it is essentially available by the action of type B ultraviolet rays on 7-dehydrocholesterol, present in the human epidermis (1). The endogen production of cholecalciferol depends on genetic factors, body compositions, rates of physical activity, and the use of sunscreen; its production is significantly higher in regions with greater latitudes, during the summer months, and around noon (2-4). It is known that the major role of vitamin D is related to the homeostasis of calcium/phosphorus and promotion of bone mineralization; however, studies conducted in the last decade suggested that vitamin deficiency may contribute to the pathogenesis of autoimmune diseases, cancer, insulin resistance, diabetes mellitus, dyslipidemia, arterial hypertension, metabolic syndrome, chronic inflammation, endothelial dysfunction, and cardiovascular disease (5-11). These findings have significantly increased interest in evaluating the serum concentration of 25 hydroxivitamin D [25(OH) D]; therefore, 25(OH) D is currently one of the most important hormones in clinical practice worldwide. Although recent studies have demonstrated that bioavailable 25(OH) D (not attached to binding protein), free 25(OH) D (not attached to binding protein/albumin), and 25(OH) D/24-25 dihydroxyvitamin D are promising diagnostic tools, the consensus continues to recommend that vitamin D status should be evaluated by measuring 25(OH) D with competitive tests (radioimmunoassay, chemiluminescence, and electrochemiluminescence) or, preferably, by high performance liquid chromatography with ultraviolet detection or together with tandem mass spectrometry (12). Independent of sex or age range, serum 25(OH) D concentrations <20 ng/mL (50 nmol/L) and <30 ng/mL (75 nmol/L) for many years were the standard to diagnose deficiency and insufficiency of vitamin D, respectively (13). Nevertheless, these cutoffs were elaborated based on Caucasian populations and no longer represented a consensus. Currently, most specialists consider that serum 25(OH) D values ≥20 ng/dL are adequate for maintaining bone health and calcium homeostasis in children and adults (14-16). However, some recognize that levels >30 ng/mL are required for the preservation of bone mineralization in specific groups, including teenagers (13), and for obtaining benefits unrelated to bone metabolism (15). The reference values used currently in Brazil to classify the levels of 25(OH) D as adequate were published by the Brazilian Clinical Pathology Society and Laboratory Medicine (BCPS/LM) and the Brazilian Endocrinology and Metabolism Society (BEMS) in 2014, which were updated in 2018 (17). Values >20 ng/mL are desirable for the healthy population up to 59 years of age, and values >30 ng/mL are recommended for the elderly (aged ≥60 years). Values >30 ng/mL are also recommended for individuals classified into certain risk groups, including pregnant women, patients with osteometabolic diseases such as sarcopenia, chronic kidney disease, diabetes mellitus, cancer, and for patients who are using glucocorticoids, anticonvulsants, and antiretroviral therapies (18). Despite the large variability of methods used for measuring and several different cutoffs, in 2007, it was estimated that approximately 1 billion people around the world presented inadequate levels of serum 25(OH) D (6). A systematic review published in 2014 analyzed 103 articles that were selected between 2003 and 2013 and concluded that vitamin D deficiency is global and mainly affects women in the Middle East. However, the authors warned that only a small number of studies were performed in children and adolescents, and that these were performed in specific populations, mainly in South America and Africa (19). In addition, in a search performed in PubMed in 11/11/2019 using the term “Vitamin D,” we identified 850 publications concerning Brazilian studies, in which only 15 estimated the prevalence of hypovitaminosis D. With a total sample of 3.338 individuals evaluated through different methodologies, the percentage of hypovitaminosis D encountered was between 8.2% and 85.7% of the Brazilian population (20-34). Considering that knowledge of vitamin D status in specific populations is important for the development of public healthcare policies associated with the prevention and treatment of hypovitaminosis, we performed the present study to evaluate the secondary data of a large sample of men and women, who were presumably healthy and of all ages, to evaluate the distribution of 25(OH) D (vitamin D profile) and prevalence of hypovitaminosis D in individuals who live in the metropolitan area of the city of Rio de Janeiro.

## PATIENTS AND METHODS

Following approval from the Research Ethics Committee of the University Hospital of the State University of Rio de Janeiro (UERJ) (registration N° 04191018.3.0000.5259), a retrospective study was conducted with the cooperation of the Endocrinology Division of the UERJ Medical School and Diagnósticos da America (DASA) diagnostics company.

From DASA’s laboratory information system, two members of DASA mined de-identified data from 80,000 consecutive patients who had 25(OH) D measurements run by DASA’s central laboratory, which is situated in Rio de Janeiro, from 1/2/2018 to 2/5/2018. Patients with chronological ages <1 year and who reported the use of therapies and/or vitamin supplements at the time of blood withdrawal were excluded, reducing our sample size to 24,483 patients.

For the analysis that required chronological age, 59 patients were excluded because of lack of data.

The analysis of 25(OH) D was performed using electrochemiluminescence (ADVIA Centaur, Siemens Healthcare Diagnostics, Tarrytown, NY, USA). The total variation coefficient and interassay variation coefficients were up to 12% and 8%, respectively. In accordance with the BCPS/LM/BEMS guidelines, we considered adequate levels of 25(OH) D >20 ng/mL and >30 ng/mL in individuals up to 59 years old and in those aged ≥60 years, respectively.

The statistical analyses of the study were performed using the statistical software ‘R’ (R’ Foundation for Statistical Computing, Vienna, Austria; URL: http://www.R-project.org/). Although the results of the analysis were not significantly affected, for better graphical visualization, we excluded extreme values from the Chauvent test. For this reason, the general sample included 24,074 patients. The skewness, kurtosis, and Shapiro-Welk tests were used to evaluate the normality of the distribution of the 25(OH) D levels in the studied population, and Bartlett and Kruskal-Wallis tests were used to evaluate the homogeneity and significance of the variables, respectively, when considering different genders and age ranges. As the hypothesis of equality for the distinct age subgroups was rejected by the Bartlett and Kruskal-Wallis tests, the Bonferroni test was then applied to identify homogeneous age groups in terms of serum levels of 25(OH) D. The A Chi-square test was used to estimate if the proportion of individuals with hypovitaminosis D differed significantly between the groups. The criterion for the determination of significance adopted in this study was 5%.

## RESULTS

Levels of 25(OH) D in 24,074 individuals aged 1-95 years (64.7% female) were analyzed.

**Figure 1:**
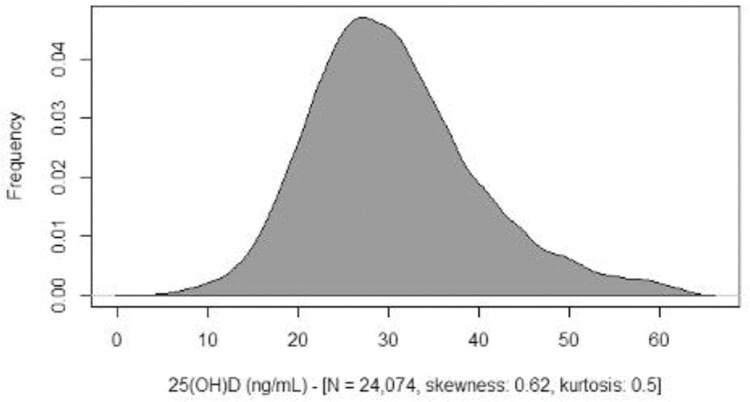
Density plot for 25-hydroxyvitamin D values (ng/mL).

Skewness, kurtosis, and Shapiro-Welk tests demonstrated that the serum levels of 25(OH) D in the studied population were slightly different from a normal distribution (Figure 1).

**Figure 2:**
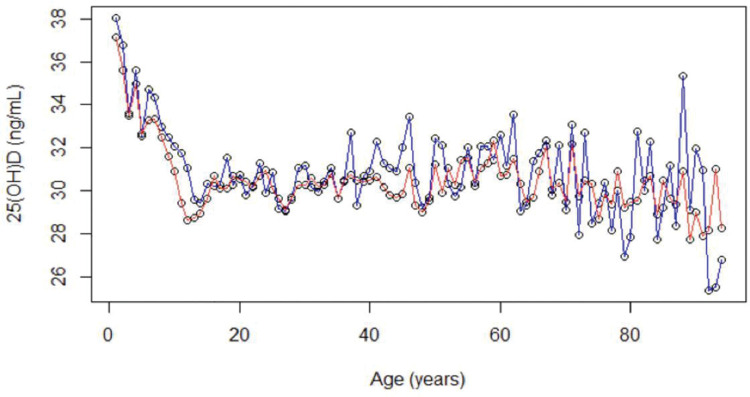
Mean values of 25-hydroxyvitamin D (ng/mL) according to sex and age. Female: red line; Male: blue line.

Descriptive curves showed that, in both sexes, the mean values of 25(OH) D decreased from the first years of life to adolescence, then slightly increased, and then tended to stabilize during adulthood (Figure 2).

The levels of 25(OH) D were higher in men at all life stages. The proportion of women with 25(OH) D levels <20 ng/mL was significantly higher than that of men in the patients aged <60 years (*p*<0.001). This difference was not significant for those aged ≥60 years (*p*=0.1588) (Figure 2).

The Bartlett test showed that there was no homogeneity in the variances when considering gender and age ranges, and the Kruskal-Wallis test indicated that, with this significant difference, the data analysis should consider the levels of 25(OH) D separately.

- 25(OH) D per age: Bartlett's K-squared=227.68, df=93, *p*=2.697e-13.
- 25(OH) D per sex: Bartlett's K-squared=13.117, df=1, *p*=0.0002927.
- 25(OH) D per age: Kruskal-Wallis K-squared=581.99, df=93, *p*<2.2e-16.
- 25(OH) D per sex: Kruskal-Wallis K-squared=80.502, df=1, *p*<2.2e-16.

The Bonferroni test identified homogenous groups for 25(OH) D levels, which were significantly different from each other (Table 1).

Based on the analysis of mean values of 25(OH) D, according to sex and to conventionally established age subgroups (Table 2), we observed a statistically significant reduction in 25(OH) D levels in the age range of 1-11 years compared with the age range of 12-18 years, in both sexes (*p*<0.001). An increase in 25(OH) D levels was also observed in the age range of 12-18 years compared with the range of 19-59 years, which was statistically significant for women (*p*<0.001). A non-significant difference in the mean 25(OH) D levels was found when comparing the age ranges of those aged 60-95 years with those aged 19-59 years.

As also shown in Table 2, 6% of female and 3.6% of male children aged <12 years presented inadequate serum levels of vitamin D (25(OH) D <20 ng/mL). This percentage increased to 13.4% and 12.6%, respectively, in female and male adolescents, and was approximately 11% in adults of both sexes <60 years of age.

The percentage of seniors with serum levels of 25(OH) D that were <20 ng/mL was 13.6% in women and 12.7% in men; however, when considering the cutoffs established by BCPS/LM/BEMS for this age range (25(OH) D <30 ng/mL), we observed that 53.2% of women aged ≥60 years and 50.6% of men aged ≥60 years had inadequate levels of 25(OH) D (Table 2).

## DISCUSSION

In this retrospective study that was accomplished by accessing the de-identified electronic database of the largest commercial laboratory in Latin America, the 25(OH) D serum levels of 24,074 presumably healthy individuals were evaluated to determine its distribution in different genders and age ranges, as well as the prevalence of hypovitaminosis D in residents of the urban metropolitan region of Rio de Janeiro, located at latitude 22°54′13- South.

We observed that, in both sexes, the levels of 25(OH) D decreased from the first years of life to adolescence, then slightly increased, and then tended to stabilize during adulthood. Furthermore, we observed that during childhood, adolescence, adulthood, and elderly life, the levels of 25(OH) D appeared to be lower in women; we also saw that the 25(OH) D mean values were homogeneous in the different age ranges and subgroups of both sexes. Children, particularly male children, presented higher 25(OH) D mean values, while female teenagers presented lower mean values.

Regarding the children and adolescents’ profiles, we verified that the results of the present study corroborated several important publications. In a populational study performed in the European continent involving 55,844 individuals, the authors observed that adolescents between 15 and 18 years of age had a higher risk of vitamin D deficiency (25(OH) D <12 ng/mL) than children aged between 1 and 14 years, adults, and even seniors (>61 years of age) (35). Additionally, in a recent study performed with 2,416 healthy children and teenagers, the authors documented that the nadir of 25(OH) D occurs between 14 and 16 years of age in male and female adolescents, respectively (36).

A reduction in serum levels of 25(OH) D in adolescents has been universally described, particularly in female adolescents, suggesting that this phase might be a risk for hypovitaminosis D presentation, which, when severe and/or for an extended period, may have negative consequences that can be relevant for both linear growth and peak bone mass (37-41). Therefore, the status of vitamin D may be an important factor to be considered in this age range (26,42).

The mechanisms responsible for an increase in the prevalence of hypovitaminosis D in the adolescent period have not yet been elucidated and may be related to biological parameters, low ingestion of foods rich in vitamin D (milk, oily fish, and specific mushrooms), reductions of sun exposure, and physical activity, all of which have been directly associated with lower levels of 25(OH) D (4,39,42). A Brazilian study conducted with 136 adolescents found that only 14.9% of this population ingested adequate quantities of vitamin D, 17.6% used topic sunscreen daily, and 27.9% did not practice regular outdoor physical activities (26). Reinforcing the importance of lifestyle, it was demonstrated that Italian teenagers who practiced outdoor activities for <3h per week presented a higher prevalence of hypovitaminosis D (16).

There is little information available concerning the levels of 25(OH) D in children. However, in a study performed in the Brazilian Southeast region, the authors observed, similarly to us, that the percentage of hypovitaminosis D is smaller in nurslings, suggesting that breastfeeding-associated vitamin D supplementation and regular sun exposure may elevate 25(OH) D levels in nurslings and young children (22).

In accordance with several publications, we observed that the mean values of 25(OH) D were lower in women at all life phases, with a significant difference in those <60 years old. This difference between genders is not well studied, and thus far, cannot be separately justified because of circulating concentrations of sexual steroids. The available studies that evaluate the correlation between serum levels of 25(OH) D, testosterone, free testosterone, estradiol, and sexual hormone binding globulin are controversial (43-47).

We also observed that female adolescents showed a decrease in 25(OH) D levels during the pubertal period and, as demonstrated by the Bonferroni test, a discrete increase after menopause, which was previously described by Katrinaki et al. (48). It is possible that the increase and decrease of tissue receptor expression to 1,25 hydroxivitamin D are linked, respectively, to increases and decreases of estrogen serum levels, and this hormonal process may contribute to vitamin D alterations (48,49). However, it is known that several other conditions may also influence 25(OH) D levels.

It is important to highlight that factors such as reduced insulin sensitivity, elevated body mass index (BMI), and increased bone turnover may be associated with the pubertal process in female adolescents and may contribute to decreases in 25(OH) D levels. In fact, Geserick et al. (36) demonstrated in their research that preceding the 25(OH) D nadir, there is a peak of bone formation and resorption markers mainly in the 3^rd^ and 4^th^ pubertal stages for girls and boys, respectively. Moreover, according to several reports in the literature (50,51), the authors reported an inverse correlation between serum levels of 25(OH) D and BMI, both in children and adolescents.

A review study published in 2009 indicated that factors such as reduction in time to sun exposure and epidermal production of vitamin D3, reduction of the expression and affinity to vitamin D receptors, lower intestinal vitamin D absorption, higher BMI, renal function deterioration, lower estrogen levels, and an increase in insulin-like growth factor 1 may all contribute to the decline of 25(OH) D levels in the elderly population (49).

Although the 25(OH) D mean value levels appear to be similar when we confront the totality of individuals over and under 60 years old, the detailed analysis of our distribution graphs suggests that values of 25 (OH) D in seniors decline with increasing age and that there is no difference in the averages between sexes in this age range. These findings, which could be confirmed by expanding our sample of older patients, are supported by the results of previous research conducted on a Mediterranean population, in which the average 25(OH) D concentrations in men and women aged >70 years were similar and significantly lower than other age range averages (48).

It is noteworthy that the mean 25(OH) D values were approximately 30 ng/mL in all subgroups evaluated in this study. Within this context, it is worth mentioning that, for the same values of 25(OH) D, parathyroid hormone concentrations were 1.5-2 times higher in senior individuals than in adolescents (52). Thus, similar serum levels of 25(OH) D may have different metabolic consequences in youth and seniors, and higher cutoff values are recognized for the elderly population.

Considering the cutoff values currently adopted in Brazil, we observed that only 6% of girls and 3.6% of boys aged <12 years showed inadequate vitamin D serum levels (25(OH) D <20 ng/mL). This percentage increased to 13.4% and 12.6%in male and female adolescents, respectively, and was nearly 11% in adults of both genders up to 59 years of age. The percentage of seniors with 25(OH) D serum levels <20 ng/mL was very similar to that observed in adolescents; however, in 53.2% of women and 50.6% of men ≥60 years of age, the serum concentrations of 25(OH) D were <30 ng/mL; therefore, they were considered inadequate for this age range.

The comparison of prevalence in the literature is not an easy task because of the large diversity of methods used for 25(OH) D dosage and the different cutoff values used in studies. Sample compositions (dimension, ethnicity, age range, comorbidities, and use of medications that interfere with absorption/action of vitamin D), geographical coordinates of the locations where the research was conducted, and seasonal differences can also impede accurate comparisons.

Data collected from studies with European populations from diverse ethnicities and residents at different latitudes showed that 8.3% and 17.7% of individuals presented levels of 25(OH) D <30 ng/mL in summer and winter, respectively. This percentage may reach 40.4% when considering Caucasians and varies according to age, with a range of 4-7% in children, 12-40% in adolescents, 9-24% in adults, and 1-8% in seniors (35).

In contrast, the results of an extensive study that involved 5,276 adults from the city of Hong Kong, located in the Northern (N) hemisphere at a similar latitude as ours (22° ° N), revealed that hypovitaminosis D (25(OH) D <30 ng/mL) was observed in 46.3% of individuals. The prevalence in patients around 20 years old was 62.5%, in those around 60 years old it was 44.5%, and in seniors aged ≥70 years the prevalence was 44.5% (53). The elevated prevalence of hypovitaminosis D in the Asian population appears to be corroborated by a recent systematic review involving 21,236 post-menopausal women, in which 99.4% of the Chinese *versus* 29% of the American subjects presented 25(OH) D concentrations <30 ng/mL (54).

Information extracted from records of the *National Institutes of Health* showed that the prevalence of hypovitaminosis D in children varies from 7% in South America to 95% in Afghanistan, and that the prevalence of hypovitaminosis D appears to be higher in women living in the Middle East (19).

Of the 15 studies regarding the prevalence of hypovitaminosis D in the Brazilian population (Table 3), only Silva et al. (31) evaluated a broad age range sample of individuals aged 14-91 years who did not receive vitamin D supplementation. Using the cutoff values of 14 and 32 ng/mL to define deficiency and insufficiency, the authors found percentages of 0.8% and 42.4%, respectively.

Araújo et al. (22) evaluated 220 adolescents aged between 15 and 19 years (mainly black women) from the city of João Pessoa (latitude 7° S), reporting that 8.2% had insufficiency (25(OH) D <30 ng/mL) and 42.7% had vitamin D deficiency (25(OH) D<20 ng/mL), a much higher prevalence than that observed in our study.

Saraiva et al. (29) analyzed 25(OH) D concentrations in 420 elderly patients from the city of São Paulo, located at latitude 23°S, in which 69.7% were female, noting that 71.2% of the institutionalized seniors (n=177) and 43.8% of the ambulatorial seniors (n =243) presented with 25(OH) D levels <20 ng/mL. Similar to our observations, the 25(OH) D averages were considerably lower in women.

Taking into consideration the variability of the cutoff values used to define hypovitaminosis D, it is possible that the use of 25(OH) D averages might be a more adequate parameter for comparing different populations. In a large study conducted with a numerous sample size of individuals who were residents at Crete Island (latitude 35° N), the averages of 25(OH) D were 19.48±9.51 ng/mL for men and 18.01±9.01 ng/mL for women, comparable to the Polish population (18.0±9.6 ng/mL), although much lower than the levels observed in our sample (48,55). The 25(OH) D average encountered in our adolescent population was also considerably higher than that reported in the Greek and Italian populations (16,48), which is equivalent to what was found in a study performed with Brazilian individuals in the rural zone of the state of São Paulo that demonstrated levels of 29.2±0.8 ng/mL (26).

The comparatively higher mean value of 25(OH) D found in our study was expected because the city of Rio de Janeiro has favorable geographical coordinates, is coastal, presents abundant sunny days in all seasons, and offers several opportunities for outdoor activities. Furthermore, the blood samples used in the study were collected during the summer period, and the estimated 25(OH) D serum concentrations were measured in presumably healthy individuals.

It is important to emphasize, however, that the present study was conducted based on retrospective information from a laboratory database, which may contain misleading reports, including those regarding the use of drugs and supplements. Additionally, with the available data, it was not possible to analyze race, BMI, physical activity, and the use of sunscreen in the selected sample.

Thus, although our results must be evaluated with proper caution, the large number of examinations included in our study, standardization of the assay for 25(OH) D dosages, and adopted criteria for both the methods and statistical analyses allow us to conclude that, in the studied population, and in accordance to the literature, the 25(OH) D mean values were higher in children and lower in adolescents and women. We also conclude that the majority of presumably healthy non-senior individuals, who resided in the metropolitan region of Rio de Janeiro, demonstrated satisfactory levels of 25(OH) D (≥20 ng/mL) during the summer months. However, more than half of the elderly population had inadequate serum 25(OH) D concentrations (<30 ng/mL). Therefore, strategies for the prevention and monitoring of hypovitaminosis D should be considered for the senior population.

## AUTHOR CONTRIBUTIONS

All authors contributed to the conception and design of the study. Material preparation and writing of the manuscript were performed by Leão LMCSM and Rodrigues BC. The first draft of the manuscript was written by Leão LMCSM and Rodrigues BC, and all authors commented on previous versions of the manuscript. Souza TSP and Hirose CK collected the data, and Dias PTP was responsible for the statistical analysis. Gehrke B contributed to the manuscript writing and translated it from Portuguese to English. Freire MDC led the project at Diagnósticos da América SA and Leão LMCSM was in charge of the entire project. All of the authors have read and approved the final version of the manuscript.

## Figures and Tables

**Table 1:** Homogenous age groups by sex for the levels of 25-hydroxivitamin D [the Bonferroni test was used to separate groups with statistically different means (*p*≤0.05)].

	n	Mean values	SD	1^st^ Quartile	Median	3^rd^ Quartile
**Female subjects**						
Group 1: Ages 1-4 years	501	34.3491	8.726559	28.4	32.9	39.7
Group 2: Ages 5-10 years	1,198	31.48205	7.981029	25.8	30.5	36.3
Group 3: Ages 11-15 years	1,087	27.91766	8.450635	22.4	26.7	32.4
Group 4: Ages 16-53 years	9,614	30.04439	9.271459	23.6	28.8	30.0
Group 5: Ages 54-95 years	3,139	30.36008	9.77338	24.2	29.6	35.8
**Total**	**15,539**					
**Male subjects**						
Group 1: Ages 1-4 years	510	35.71765	9.155856	29.2	35.0	41.5
Group 2: Ages 5-11 years	1.433	32.93936	8.683551	26.7	31.8	38.0
Group 3: Ages 12-95 years	6.533	30.63023	9.676679	23.9	29.6	35.9
**Total**	**8,476**					

n: sample size; SD: standard deviation.

**Table 2:** Means, medians, and hypovitaminosis D percentages by sex and age group.

	Mean	SD	Median	25^th^ Quartile	75^th^ Quartile	%
**From 1 to 11 (years old)**						
**Female (n=1928)**	31.7	8.4	30.7	25.8	36.8	
25(OH) D <20 ng/mL	17.5	2.3	18.1	16.5	19.3	6.0
**Male (n=1943)**	33.7	8.9	32.4	27.2	39.0	
25(OH) D <20 ng/mL	17.8	2.0	18.3	17.3	19.2	3.6
**From 12 to 18 (years old)**						
**Female (n=1429)**	28.9	8.8	27.8	23.0	33.7	
25(OH) D <20 ng/mL	16.7	2.8	17.6	15.2	18.7	13.4
**Male (n=1092)**	30.3	9.8	29.2	23.6	35.6	
25(OH) D <20 ng/mL	16.2	3.3	17.4	14.2	18.9	12.6
**From 19 to 59 (years old)**						
**Female (n=9918)**	30.2	9.3	28.8	23.7	35.4	
25(OH) D <20 ng/mL	16.8	2.7	17.5	15.6	18.9	11.3
**Male (n=4409)**	30.7	9.6	29.6	24.1	35.9	
25(OH) D <20 ng/mL	16.7	2.8	17.3	15.0	18.9	11.0
**From 60 to 95 (years old)**						
**Female (n=2264)**	30.0	9.8	29.3	23.8	35.4	
25(OH) D <20 ng/mL	15.1	3.8	16.1	12.2	18.4	13.6
25(OH) D <30 ng/mL	22.9	5.5	24.2	19.8	27.2	53.2
**Male (n=1032)**	30.6	9.9	29.8	23.8	35.8	
25(OH) D <20 ng/mL	15.9	3.4	16.7	14.9	18.6	12.7
25(OH) D <30 ng/mL	23.1	5.1	23.9	19.8	27.2	50.6

25(OH) D: 25 hydroxivitamin D; n: sample size by age and sex; SD: standard deviation; %: percentage.

**Table 3:** Brazilian studies regarding the prevalence of hypovitaminosis D.

	Place of Study	Study population	n	Assay 25(OH)	% Hypovitaminosis D
Premaor et al. (27)	Porto Alegre Latitude: 30°S	Males (54.3%) and women >65 years of age, light skinned (58.8%) Most patients had been recently hospitalized	81	Radioimmunoassay	77.8% <20 ng/mL
Saraiva et al. (29)	São Paulo Latitude: 23°S	Institutionalized seniors (70.6% female; mean age 76.6 years) and no institutionalized seniors (69.1% female; mean age 79.1 years)	420	Radioimmunoassay	Institutionalized: 71.2% <20 ng/mL Outpatients: 43.8% <20 ng/mL
Scalco et al. (30)	Porto Alegre Latitude: 30°S	Seniors (>65 years of age) residing in nursing homes	102	Chemiluminescence	85.7% <20 ng/mL
Silva et al. (31)	Belo Horizonte Latitude: 19°S	Women (91.6%) and men (14-91 years of age, mean age 58.87 years)	132	High performance liquid chromatography	0.8% <14 ng/mL 42.4% <32 ng/mL
Peters et al. (26)	Rural Town Sâo Paulo Latitude: 23°S	Children and adolescents (6-20 years of age)	136	Radioimmunoassay	60.0% <30 ng/mL
Souto et al. (33)	Rio de Janeiro Latitude: 22°S	Patients with systemic lupus erythematosus (94.3% female; 67.9% caucasian)	159	High performance liquid chromatography	8.2% <20 ng/mL
Santos et al. (28)	Curitiba Latitude: 25°S Porto Alegre Latitude: 30°S	Apparently healthy girls (7-18 years of age)	234	Radioimmunoassay	36.3% <20 ng/mL
Cabral et al. (21)	Recife Latitude: 8°S	Male and female (69.4±6.5 years of age; 66.7% had dark skin)	284	Electrochemiluminescent competitive immunoassay	66.7% <30 ng/mL
Ferreira et al. (18)	Maceió Latitude: 9°S	HIV-infected adults (≥20 years of age)	113	Chemiluminescence	23.9% <30 ng/mL
Lopes et al. (24)	Brasília Latitude: 15°S	Infertile women and controls (21-47 years of age)	369	Chemiluminescence	32.0% <20 ng/mL (infertile and control patients)
Araújo et al. (22)	João Pessoa Latitude: 7°S	Post-pubertal adolescents (15-19 years of age)	220	Chemiluminescence	8.2 <30 ng/mL 42.7% <20 ng/mL
Chrisostomo et al. (20)	Curitiba Latitude: 25°S	Pregnant women (18-40 years of age)	520	Microparticle chemiluminescence	43.7% <20 ng/mL
Peçanha et al. (25)	Viçosa Latitude: 20°S	Children, mostly biracial or black boys (5.8±4.6 years of age)	124	Chemiluminescence	57.3% <20 ng/mL
Vivan et al. (34)	Porto Alegre Latitude: 30°S	Pre-bariatric patients (76.6% female; mean age 44.9 years)	291	Chemiluminescence	55.3% <20 ng/mL
Sousa et al. (32)	Natal Latitude: 5°S	Seniors (>60 years of age) from nine nursing homes	153	Chemiluminescence	71.2% <30 ng/mL

n: sample size.

## References

[1] Thacher TD, Clarke BL (2011). Vitamin D insufficiency. Mayo Clin Proc.

[2] Saraff V, Shaw N (2016). Sunshine and vitamin D. Arch Dis Child.

[3] DeLuca HF (2004). Overview of general physiologic features and functions of vitamin D. Am J Clin Nutr.

[4] Valtueãa J, González-Gross M, Huybrechts I, Breidenassel C, Ferrari M, Mouratidou T (2013). Factors associated with vitamin D deficiency in European adolescents: the HELENA study. J Nutr Sci Vitaminol (Tokyo).

[5] Casella CB, Seguro LP, Takayama L, Medeiros D, Bonfa E, Pereira RM (2012). Juvenile onset systemic lupus erythematosus: a possible role for vitamin D in disease status and bone health. Lupus.

[6] Holick MF (2007). Vitamin D deficiency. N Engl J Med.

[7] Al-Daghri NM, Alkharfy KM, Al-Othman A, El-Kholie E, Moharram O, Alokail MS (2012). Vitamin D supplementation as an adjuvant therapy for patients with T2DM: an 18-month prospective interventional study. Cardiovasc Diabetol.

[8] Min B (2013). Effects of vitamin D on blood pressure and endothelial function. Korean J Physiol Pharmacol.

[9] Chiu KC, Chu A, Go VL, Saad MF (2004). Hypovitaminosis D is associated with insulin resistance and beta cell dysfunction. Am J Clin Nutr.

[10] Pittas AG, Lau J, Hu FB, Dawson-Hughes B (2007). The role of vitamin D and calcium in type 2 diabetes. A systematic review and meta-analysis. J Clin Endocrinol Metab.

[11] Mascitelli L, Goldstein MR, Pezzetta F (2010). [Vitamin D deficiency and cardiovascular diseases]. Recenti Prog Med.

[12] Herrmann M, Farrell CL, Pusceddu I, Fabregat-Cabello N, Cavalier E (2017). Assessment of vitamin D status - a changing landscape. Clin Chem Lab Med.

[13] Holick MF, Binkley NC, Bischoff-Ferrari HA, Gordon CM, Hanley DA, Heaney RP (2011). Evaluation, treatment, and prevention of vitamin D deficiency: an Endocrine Society clinical practice guideline. J Clin Endocrinol Metab.

[14] Munns CF, Shaw N, Kiely M, Specker BL, Thacher TD, Ozono K (2016). Global Consensus Recommendations on Prevention and Management of Nutritional Rickets. J Clin Endocrinol Metab.

[15] Płudowski P, Karczmarewicz E, Bayer M, Carter G, Chlebna-Sokół D, Czech-Kowalska J (2013). Practical guidelines for the supplementation of vitamin D and the treatment of deficits in Central Europe - recommended vitamin D intakes in the general population and groups at risk of vitamin D deficiency. Endokrynol Pol.

[16] Vierucci F, Del Pistoia M, Fanos M, Erba P, Saggese G (2014). Prevalence of hypovitaminosis D and predictors of vitamin D status in Italian healthy adolescents. Ital J Pediatr.

[17] Maeda SS, Borba VZ, Camargo MB, Silva DM, Borges JL, Bandeira F (2014). Recommendations of the Brazilian Society of Endocrinology and Metabology (SBEM) for the diagnosis and treatment of hypovitaminosis D. Arq Bras Endocrinol Metab.

[18] Ferreira CES, Maeda SS, Batista MC, Castro ML, Vasconcellos LS, Madeira M (2018). Posicionamento Oficial da Sociedade Brasileira de Patologia Clínica/Medicina Laboratorial e da Sociedade Brasileira de Endocrinologia e Metabologia- Intervalos de Referência da Vitamina D - 25(OH)D.

[19] Palacios C, Gonzalez L (2014). Is vitamin D deficiency a major global public health problem?. J Steroid Biochem Mol Biol.

[20] Chrisostomo KR, Skare TL, Kulak J, Urbanetz AA, Chrisostomo ER, Nisihara R (2018). The prevalence and clinical associations of hypovitaminosis D in pregnant women from Brazil. Int J Gynaecol Obstet.

[21] Cabral MA, Borges CN, Maia JM, Aires CA, Bandeira F (2013). Prevalence of vitamin D deficiency during the summer and its relationship with sun exposure and skin phototype in elderly men living in the tropics. Clin Interv Aging.

[22] Santos Araújo EPD, Queiroz DJM, Neves JPR, Lacerda LM, Gonçalves MDCR, Carvalho AT (2017). Prevalence of hypovitaminosis D and associated factors in adolescent students of a capital of northeastern Brazil. Nutr Hosp.

[23] Ferreira SM, Lima MH, Omena AL, Canuto JM, Canuto VM, Morais TM (2016). Prevalence of hypovitaminosis D and its association with oral lesions in HIV-infected Brazilian adults. Rev Soc Bras Med Trop.

[24] Lopes VM, Lopes JR, Brasileiro JP, Oliveira I, Lacerda RP, Andrade MR (2017). Highly prevalence of vitamin D deficiency among Brazilian women of reproductive age. Arch Endocrinol Metab.

[25] Peçanha MB, Freitas RB, Moreira TR, Silva LS, Oliveira LL, Cardoso SA (2019). Prevalence of vitamin D deficiency and its relationship with factors associated with recurrent wheezing. J Bras Pneumol.

[26] Peters BS, dos Santos LC, Fisberg M, Wood RJ, Martini LA (2009). Prevalence of vitamin D insufficiency in Brazilian adolescents. Ann Nutr Metab.

[27] Premaor MO, Alves GV, Crossetti LB, Furlanetto TW (2004). Hyperparathyroidism secondary to hypovitaminosis D in hypoalbuminemic is less intense than in normoalbuminemic patients: a prevalence study in medical inpatients in southern Brazil. Endocrine.

[28] Santos BR, Mascarenhas LP, Satler F, Boguszewski MC, Spritzer PM (2012). Vitamin D deficiency in girls from South Brazil: a cross-sectional study on prevalence and association with vitamin D receptor gene variants. BMC Pediatr.

[29] Saraiva GL, Cendoroglo MS, Ramos LR, Araújo LM, Vieira JG, Maeda SS (2007). Prevalência da deficiência, insuficiência de vitamina D e hiperparatiroidismo secundário em idosos institucionalizados e moradores na comunidade da cidade de São Paulo, Brasil. Arq Bras Endocrinol Metab.

[30] Scalco R, Premaor MO, Fröehlich PE, Furlanetto TW (2008). High prevalence of hypovitaminosis D and secondary hyperparathyroidism in elders living in nonprofit homes in South Brazil. Endocrine.

[31] Silva BC, Camargos BM, Fujii JB, Dias EP, Soares MM (2008). Prevalência de deficiência e insuficiência de vitamina D e sua correlação com PTH, marcadores de remodelação óssea e densidade mineral óssea, em pacientes ambulatoriais. Arq Bras Endocrinol Metabol.

[32] Sousa SES, Sales MC, Araújo JRT, Sena-Evangelista KCM, Lima KC, Pedrosa LFC (2019). High Prevalence of Hypovitaminosis D in Institutionalized Elderly Individuals is Associated with Summer in a Region with High Ultraviolet Radiation Levels. Nutrients.

[33] Souto M, Coelho A, Guo C, Mendonça L, Argolo S, Papi J (2011). Vitamin D insufficiency in Brazilian patients with SLE: prevalence, associated factors, and relationship with activity. Lupus.

[34] Vivan MA, Kops NL, Fülber ER, de Souza AC, Fleuri MASB, Friedman R (2019). Prevalence of Vitamin D Depletion, and Associated Factors, among Patients Undergoing Bariatric Surgery in Southern Brazil. Obes Surg.

[35] Cashman KD, Dowling KG, Skrabáková Z, Kiely M, Lamberg-Allardt C, Durazo-Arvizu RA (2015). Standardizing serum 25-hydroxyvitamin D data from four Nordic population samples using the Vitamin D Standardization Program protocols: Shedding new light on vitamin D status in Nordic individuals. Scand J Clin Lab Invest.

[36] Geserick M, Vogel M, Eckelt F, Schlingmann M, Hiemisch A, Baber R (2020). Children and adolescents with obesity have reduced serum bone turnover markers and 25-hydroxyvitamin D but increased parathyroid hormone concentrations - Results derived from new pediatric reference ranges. Bone.

[37] Ganji V, Zhang X, Tangpricha V (2012). Serum 25-hydroxyvitamin D concentrations and prevalence estimates of hypovitaminosis D in the U.S. population based on assay-adjusted data. J Nutr.

[38] Whiting SJ, Langlois KA, Vatanparast H, Greene-Finestone LS (2011). The vitamin D status of Canadians relative to the 2011 Dietary Reference Intakes: an examination in children and adults with and without supplement use. Am J Clin Nutr.

[39] González-Gross M, Valtueãa J, Breidenassel C, Moreno LA, Ferrari M, Kersting M (2012). Vitamin D status among adolescents in Europe: the Healthy Lifestyle in Europe by Nutrition in Adolescence study. Br J Nutr.

[40] Kim SH, Oh MK, Namgung R, Park MJ (2014). Prevalence of 25-hydroxyvitamin D deficiency in Korean adolescents: association with age, season and parental vitamin D status. Public Health Nutr.

[41] Siddiqui AM, Kamfar HZ (2007). Prevalence of vitamin D deficiency rickets in adolescent school girls in Western region, Saudi Arabia. Saudi Med J.

[42] Smith TJ, Tripkovic L, Lanham-New SA, Hart KH (2018). Vitamin D in adolescence: evidence-based dietary requirements and implications for public health policy. Proc Nutr Soc.

[43] Zhao D, Ouyang P, de Boer IH, Lutsey PL, Farag YM, Guallar E (2017). Serum vitamin D and sex hormones levels in men and women: The Multi-Ethnic Study of Atherosclerosis (MESA). Maturitas.

[44] Rafiq R, van Schoor NM, Sohl E, Zillikens MC, Oosterwerff MM, Schaap L (2016). Associations of vitamin D status and vitamin D-related polymorphisms with sex hormones in older men. J Steroid Biochem Mol Biol.

[45] Wang N, Han B, Li Q, Chen Y, Chen Y, Xia F (2015). Vitamin D is associated with testosterone and hypogonadism in Chinese men: Results from a cross-sectional SPECT-China study. Reprod Biol Endocrinol.

[46] Chang EM, Kim YS, Won HJ, Yoon TK, Lee WS (2014). Association between sex steroids, ovarian reserve, and vitamin D levels in healthy nonobese women. J Clin Endocrinol Metab.

[47] Pop LC, Shapses SA, Chang B, Sun W, Wang X (2015). Vitamin D-Binding Protein In Healthy Pre- And Postmenopausal Women: Relationship With Estradiol Concentrations. Endocr Pract.

[48] Katrinaki M, Kampa M, Margioris A, Castanas E, Malliaraki N (2016). Vitamin D levels in a large Mediterranean cohort: reconsidering normal cut-off values. Hormones (Athens).

[49] Oudshoorn C, van der Cammen TJ, McMurdo ME, van Leeuwen JP, Colin EM (2009). Ageing and vitamin D deficiency: effects on calcium homeostasis and considerations for vitamin D supplementation. Br J Nutr.

[50] Snijder MB, van Dam RM, Visser M, Deeg DJ, Dekker JM, Bouter LM (2005). Adiposity in relation to vitamin D status and parathyroid hormone levels: a population-based study in older men and women. J Clin Endocrinol Metab.

[51] Earthman CP, Beckman LM, Masodkar K, Sibley SD (2012). The link between obesity and low circulating 25-hydroxyvitamin D concentrations: considerations and implications. Int J Obes (Lond).

[52] Soares LM, Pedrosa W, Elói-Santos SM, Vasconcellos LS (2017). 25-Hydroxyvitamin D threshold values should be age-specific. Clin Chem Lab Med.

[53] Leung RY, Cheung BM, Nguyen US, Kung AW, Tan KC, Cheung CL (2017). Optimal vitamin D status and its relationship with bone and mineral metabolism in Hong Kong Chinese. Bone.

[54] Valladares T, Simões R, Bernardo W, Schmitt ACB, Cardoso MRA, Aldrighi JM (2019). Prevalence of hypovitaminosis D in postmenopausal women: a systematic review. Rev Assoc Med Bras (1992).

[55] Płudowski P, Ducki C, Konstantynowicz J, Jaworski M (2016). Vitamin D status in Poland. Pol Arch Med Wewn.

